# Ki-67 Proliferation Index Improves the Prognostic Stratification Accuracy of International Staging System in Newly Diagnosed Multiple Myeloma

**DOI:** 10.3390/cancers18203312

**Published:** 2026-10-14

**Authors:** Xiaoqi Qin, Jingliang Zhao, Qirui Bai, Qile Qiu, Shengtao Li, Yifan Zhang, Lin Zhang, Yanping Ma

**Affiliations:** 1 Department of Hematology, The Second Hospital of Shanxi Medical University, Taiyuan 030000, China; 2 Fifth Tianjin Central Hospital, Tianjin 300450, China

**Keywords:** multiple myeloma, Ki-67, ISS, prognosis

## Abstract

**Objective**: To investigate the clinical characteristics and prognostic significance of distinct Ki-67 proliferation indices in patients with newly diagnosed multiple myeloma (NDMM). **Methods**: A retrospective analysis of clinical data from 307 NDMM patients admitted to the Second Hospital of Shanxi Medical University from January 2015 to December 2023. Patients were stratified into two cohorts based on Ki-67 proliferation index: Ki-67 > 10% and Ki-67 ≤ 10%. **Results**: Among the 307 enrolled NDMM patients, 199 (64.82%, 199/307) were assigned to the Ki-67 ≤ 10% group. The median progression-free survival (mPFS) was 45.52 months (95% CI: 39.40–51.66) in the Ki-67 ≤ 10% group, compared with 25.81 months (95% CI: 20.61–31.00) in the Ki-67 > 10% group, with a statistically significant difference in PFS between the two cohorts (*p* < 0.001). Between these two groups, the median Overall Survival (mOS) reached 61.61 months (95% CI: 54.48–68.74) in the Ki-67 ≤ 10% group and 34.78 months (95% CI: 28.89–40.66) in the Ki-67 > 10% group, with a statistically significant difference in OS between the two cohorts (*p* < 0.001). Multivariate Cox proportional hazards regression analysis demonstrated that the Ki-67 proliferation index served as an independent prognostic factor for both PFS (HR:1.227; 95% CI: 1.097–1.743; *p* = 0.043) and OS (HR:1.192; 95% CI: 1.169–1.847; *p* = 0.048) in patients with NDMM. Subsequently, patients with NDMM were stratified into three risk groups based on the combination of International Staging System (ISS) stage and Ki-67 proliferation index. A total of 103 patients (33.55%) were classified into group I, 138 patients (44.95%) into group II, and 66 patients (21.50%) into group III. Survival analyses revealed significant differences in survival outcomes across the three combined staging groups. The mPFS was 49.74 months (95% CI: 28.81–63.19) in group I, 35.00 months (95% CI: 24.82–45.18) in group II, and 17.00 months (95% CI: 13.94–20.06) in group III patients, with a statistically significant inter-group difference in PFS (*p* = 0.011). Meanwhile, the mOS of the three groups was 71.38 months (95% CI: 63.59–79.17), 59.51 months (95% CI: 51.74–67.28), and 26.64 months (95% CI: 22.31–30.98), respectively. There was an obvious significant difference in OS among the three groups (*p* < 0.001). **Conclusions**: NDMM patients with Ki-67 proliferation index > 10% have a significantly worse prognosis than those with Ki-67 ≤ 10%. A high Ki-67 index (>10%) is identified as an independent adverse prognostic parameter for NDMM. Furthermore, the integration of Ki-67 proliferation index with ISS staging can substantially enhance the accuracy of prognostic risk stratification in patients with NDMM.

## Simple Summary

Multiple myeloma is a highly heterogeneous hematological malignancy. The proliferative potential of tumor cells is closely associated with poor prognosis, and Ki-67 is an important parameter reflecting the proliferative capacity of tumor cells. This study aims to further improve the accuracy of the International Staging System for multiple myeloma. This retrospective study analyzed data from 307 patients with newly diagnosed multiple myeloma. It found that patients with a Ki-67 proliferation index > 10% had significantly shorter progression-free survival and overall survival, and Ki-67 served as an independent adverse prognostic factor for newly diagnosed multiple myeloma. A three-tier risk-stratification model constructed by combining the Ki-67 index with International Staging System could identify high-risk patients within International Staging System stage I and low-risk patients within International Staging System stage III. This model effectively compensates for the limitations of the International Staging System alone and improves the accuracy of prognostic risk stratification for newly diagnosed multiple myeloma patients. Based on routine risk factors, the model is convenient for popularization and application in real-world clinical practice.

## 1. Introduction

Multiple myeloma (MM) is a hematological malignancy characterized by the clonal expansion of aberrant plasma cells within the bone marrow, the vast majority of which secrete monoclonal immunoglobulins or free light chains. Tumor cells of MM exhibit highly heterogeneous biological features. Despite substantial advances in therapeutic strategies for MM over the past decades, the prognosis of MM patients remains markedly variable: overall survival ranges from only a few months to more than a decade among different individuals [1]. A variety of tumor biomarkers and prognostic staging systems have been extensively applied for risk stratification in MM, enabling individualized therapeutic strategies for patients with different risk profiles. Classic and representative staging frameworks encompass the ISS, which was developed by the International Myeloma Working Group and relies only on serum beta-2-microglobulin and albumin for risk stratification. The Revised International Staging System (R-ISS), which incorporates high-risk cytogenetic abnormalities and lactate dehydrogenase (LDH) on the basis of ISS, was proposed to improve risk stratification for MM. The Second Revision of the International Staging System (R2-ISS) was developed by the European Myeloma Network to further refine risk stratification. The Mayo Additive Staging System (MASS), established at the Mayo Clinic, is an additive scoring model for NDMM. The Myeloma Prognostic Score System (MPSS) is a weighted prognostic model developed and validated in Chinese and Western myeloma cohorts.

However, each of these conventional staging systems has inherent deficiencies and cannot accurately distinguish the heterogeneous prognostic risks among MM patients. For example, the ISS fails to incorporate cytogenetic aberrations of clonal plasma cells. Although the R-ISS integrates high-risk cytogenetic lesions, it assigns equal weight to all cytogenetic abnormalities without hierarchical stratification, which has driven the establishment of the R2-ISS and MASS staging systems. Despite these optimized staging tools, a considerable proportion of patients with high-risk MM cannot be reliably identified at initial diagnosis, and such patients are only retrospectively defined as functionally high-risk after disease relapse [2].

Clonal plasma cells generally exhibit low proliferative activity in patients with NDMM. The proliferative potential of malignant plasma cells increases significantly during disease progression and relapse. Elevated plasma cell proliferative activity has been well recognized as a robust adverse prognostic factor for plasma cell neoplasms [3].

Ki-67, a nuclear proliferation-associated antigen, is expressed during the G1, S, G2, and M phases of the cell cycle, whereas quiescent cells in the G0 phase lack Ki-67 expression. Elevated Ki-67 levels strongly correlate with enhanced proliferative activity of malignant cells and serve as a marker of inferior prognosis across a broad spectrum of human malignancies. In MM, accumulating evidence has demonstrated that high Ki-67 expression correlates with unfavorable clinical outcomes. However, most existing clinical guidelines have not incorporated Ki-67 into well-established risk stratification systems for MM. To further elucidate the prognostic significance of Ki-67 expression in NDMM, this retrospective study investigated the correlations of bone marrow immunohistochemical Ki-67 index with baseline clinical profiles, biological characteristics of clonal plasma cells, treatment responses, and survival outcomes in NDMM patients treated at our center in recent years. We also aimed to restratify NDMM patients by combining the Ki-67 index with the ISS and further explored whether the incorporation of Ki-67 could improve the prognostic efficacy of the widely used ISS risk stratification model for NDMM.

## 2. Materials and Methods

### 2.1. Patients

We retrospectively enrolled 307 patients with NDMM who presented to the Second Hospital of Shanxi Medical University between January 2015 and December 2023. All patients fulfilled the diagnostic criteria established by the International Myeloma Working Group (IMWG). Clinical data retrieved from medical records during prior therapeutic management were collected, and patients were dichotomized into two cohorts based on Ki-67 index cutoffs: Ki-67 > 10% and Ki-67 ≤ 10%. Baseline parameters collected for all NDMM participants included sex, age, routine blood test results, hepatic and renal function, serum electrolytes, LDH, serum and urine M-protein isotypes, cytogenetic abnormalities, presence or absence of extramedullary disease, and post-treatment hematologic response.

This retrospective study was approved by the Medical Ethics Committee of the Second Hospital of Shanxi Medical University (Approval No. [2026]YX-236).

### 2.2. Methods

#### 2.2.1. Treatment Regimens

The main induction regimens consisted of BD (bortezomib plus dexamethasone), BCD (bortezomib, cyclophosphamide plus dexamethasone), and VRD (bortezomib, lenalidomide plus dexamethasone). The detailed dosing schedules were as follows: bortezomib at 1.3 mg/m^2^ on days 1, 4, 8, and 11; cyclophosphamide at 300 mg/m^2^ on days 1, 8, and 15; lenalidomide 25 mg once daily on days 1–21, with dose adjustments according to creatinine clearance for patients with renal insufficiency; and dexamethasone 20 mg on days 1, 2, 4, 5, 8, 9, 11, and 12. Following 3 to 4 cycles of induction therapy, autologous stem cell transplantation (ASCT) was performed based on the patients’ physical condition and personal willingness. Patients who did not undergo ASCT continued the initial induction treatment for a total of 8 to 12 cycles, followed by 1 to 2 years of maintenance therapy with bortezomib- or lenalidomide-based regimens.

#### 2.2.2. Clinical Endpoint Definitions

Progression-free survival (PFS) was defined as the time from the initial diagnosis of multiple myeloma to the first recorded disease progression or relapse, all-cause death, or the date of follow-up censoring. Overall survival (OS) was defined as the time from initial diagnosis until all-cause death or censoring at the last follow-up.

#### 2.2.3. Ki-67 Quantification

Ki-67 immunohistochemical staining was performed on formalin-fixed paraffin-embedded bone marrow specimens. The anti-Ki-67 antibody was obtained from Leica Biosystems (catalog number NCL-KI67, Wetzlar, Germany). All immunohistochemistry experiments were completed by certified pathotechnologists in the department of pathology. Strict internal quality control was implemented during IHC staining, including positive and negative tissue controls for each batch of experiments. In this retrospective study, the Ki-67 proliferative index was manually evaluated by experienced pathologists under light microscopy. Tumor nuclei with any Ki-67 staining were regarded as positive, and 500 infiltrated plasma cells were counted in hot-spot areas to calculate the Ki-67 index.

#### 2.2.4. Follow-Up and Survival Analysis

All enrolled patients were followed up through telephone interviews, outpatient reexaminations, and review of inpatient medical records. The median follow-up time was 54.8 months (range: 2.6–129 months), and the final follow-up cutoff was set in June 2025. OS was defined as the time from the initial diagnosis of multiple myeloma to all-cause death or loss to follow-up. PFS was defined as the time from initial diagnosis to disease progression, death, or loss to follow-up.

#### 2.2.5. Statistical Analysis

All statistical analyses were conducted using SPSS 25.0 software. The chi square (χ^2^) test was used to compare categorical variables between groups. For continuous variables, the independent samples *t*-test or one-way analysis of variance (ANOVA) was adopted for intergroup comparison of mean values. Survival curves were plotted using the Kaplan–Meier method, and the log rank test was utilized to evaluate differences in survival outcomes. Multivariate prognostic analysis was performed via the Cox proportional hazards regression model. A two-tailed *p* value less than 0.05 was considered statistically significant.

## 3. Results

### 3.1. Patient Characteristics

A total of 307 patients with NDMM were enrolled in this study, including 181 males and 126 females with a median diagnostic age of 61 years (range, 37–86 years). In terms of M-protein isotypes, 134 patients (43.65%) had IgG-type myeloma, 66 (21.50%) had IgA-type disease, 15 (4.89%) had IgD-type myeloma, 82 (26.71%) presented with light-chain myeloma, 9 (2.93%) had non-secretory myeloma, and 1 (0.33%) had biclonal myeloma. According to the According to the Durie–Salmon (DS) staging system, 13 patients (4.23%) were stage I, 26 (8.47%) stage II, and 268 (87.30%) stage III. Based on the ISS, 27 patients (8.79%) were classified as stage I, 105 (34.20%) as stage II, and 175 (57.00%) as stage III.

### 3.2. Comparison of Baseline Characteristics Between Patients Stratified by Ki-67 Index (Ki-67 > 10% Versus Ki-67 ≤ 10%)

Baseline parameters including age, sex, M-protein isotype, Durie–Salmon (DS) stage, ISS stage, serum albumin, LDH, serum creatinine, serum calcium, and bone marrow plasma cell percentage were comparable between the Ki-67 > 10% and Ki-67 ≤ 10% groups, with no statistically significant intergroup differences (*p* ≥ 0.05). Significant between-group disparities were observed in hemoglobin (HGB), platelet (PLT) counts, and the incidence of del(17p) (all *p* < 0.05). Specifically, patients with Ki-67 ≤ 10% exhibited higher hemoglobin and platelet levels, alongside a lower prevalence of del(17p), relative to those with Ki-67 > 10% (Table 1).

### 3.3. Survival Analysis

**Figure 1:**
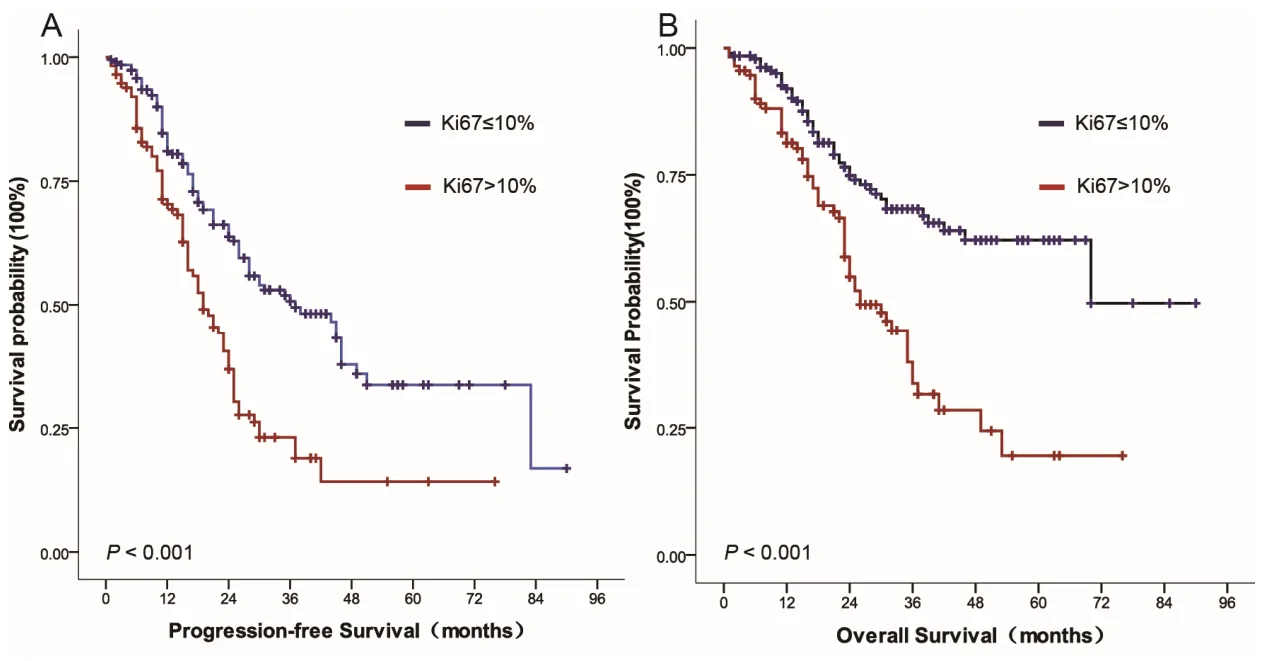
Kaplan–Meier survival curves for progression-free survival (PFS) and overall survival (OS) comparing Ki 67 ≤ 10% and Ki 67 > 10% groups. (**A**) PFS. (**B**) OS.

Kaplan–Meier survival analysis was performed on all 307 NDMM patients with complete follow-up data, with a median follow-up duration of 54.8 months. The mPFS for the entire cohort of 307 NDMM patients was 38.45 (95% CI:33.84–43.06) months. When patients were stratified by a Ki-67 cutoff of 10%, the mPFS reached 45.52 (95% CI:39.40–51.66) months in the Ki-67 ≤ 10% group versus 25.81 (95% CI: 20.61–31.00) months in the Ki-67 > 10% group, and the difference in mPFS between the two cohorts was statistically significant (*p* < 0.001; Figure 1A). The mOS in all 307 patients was 52.60 (95% CI: 47.03–58.16) months. Stratification by the Ki-67 cutoff of 10% also revealed distinct OS outcomes: the mOS was 61.61 (95% CI: 54.48–68.74) months for patients with Ki-67 ≤ 10%, compared with 34.78 (95% CI: 28.89–40.66) months for those with Ki-67 > 10%. The difference in mOS between these two groups was statistically significant (*p* < 0.001; Figure 1B).

### 3.4. Multivariate Analysis Using the Cox Proportional Hazards Model

Univariate Cox regression was first performed to screen candidate predictors of PFS. Univariate analysis identified the ISS stage, del(17p), elevated LDH, extramedullary disease, thrombocytopenia, and Ki-67 grouping as significant prognostic factors for PFS. All six abovementioned variables were subsequently incorporated into the multivariate Cox regression model. Multivariate analysis demonstrated ISS stage [HR = 1.384 (95% CI: 1.127–1.978); *p* = 0.041], del(17p) [HR = 1.517 (95% CI: 1.248–2.165); *p* = 0.013], and Ki-67 grouping [HR = 1.227 (95% CI: 1.097–1.743); *p* = 0.043] as independent adverse prognostic factors for PFS.

Univariate Cox regression was conducted to screen potential predictors of OS. ISS stage, del(17p), elevated LDH, extramedullary disease, and Ki-67 grouping were identified as significant univariate prognostic factors for OS. These five variables were entered into the multivariate Cox proportional hazards regression model. Multivariate analysis revealed that elevated LDH [HR = 1.263 (95% CI: 1.102–1.569); *p* = 0.007], extramedullary disease [HR = 1.217 (95% CI: 1.182–1.478); *p* = 0.017], and Ki-67 grouping [HR = 1.192 (95% CI: 1.169–1.847); *p* = 0.048] constituted independent adverse prognostic factors for OS.

### 3.5. Impact of Ki-67 Proliferation Index on the Prognostic Performance of ISS Staging

**Figure 2:**
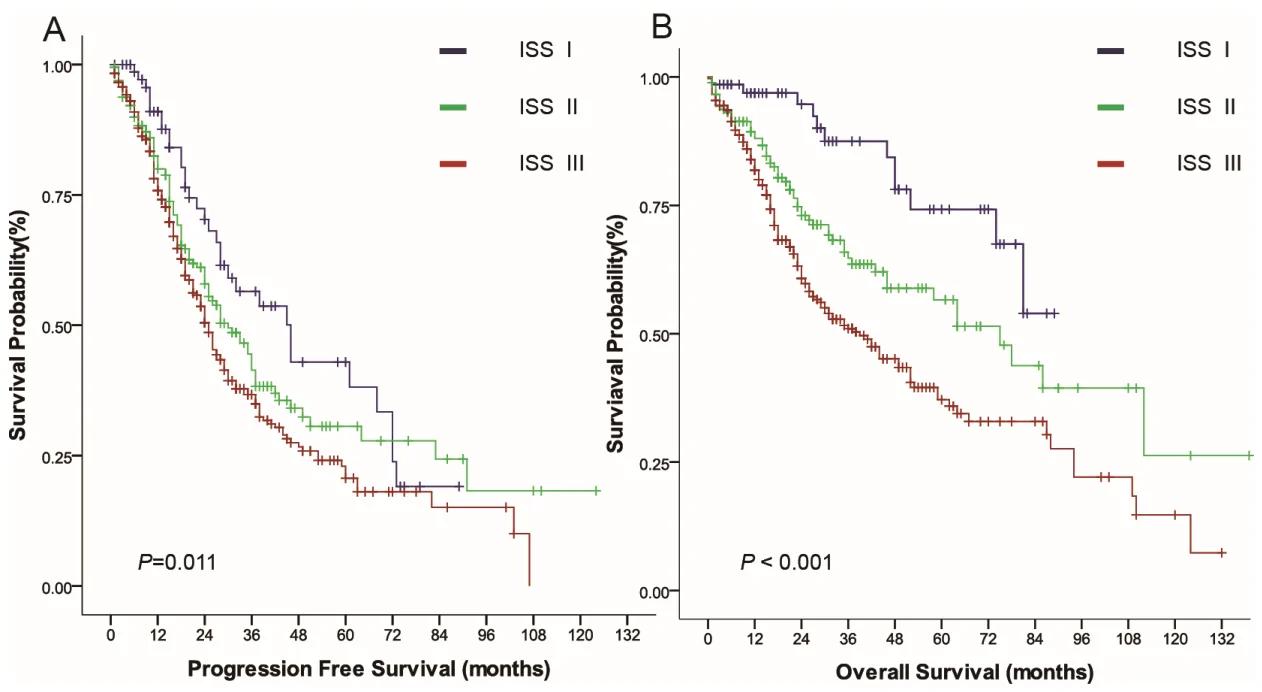
Kaplan–Meier survival curves for progression-free survival (PFS) and overall survival (OS) comparing ISS I, II and III groups. (**A**) PFS. (**B**) OS.

The PFS and OS of all 307 enrolled patients with NDMM were evaluated according to the ISS. The mPFS was 45.00 months (95% CI: 20.60–69.38) for ISS stage I, 31.00 months (95% CI: 17.92–44.07) for stage II, and 24.00 months (95% CI: 20.50–27.49) for stage III, with a statistically significant difference in PFS across the three groups (*p* = 0.011; Figure 2A). Correspondingly, the mOS reached 89.00 months (95% CI: 58.02–102.48) in ISS stage I, 75.00 months (95% CI: 55.20–94.79) in stage II, and 39.00 months (95% CI: 28.18–49.81) in stage III. The intergroup difference in OS among the three ISS strata was statistically significant (*p* < 0.001; Figure 2B).

**Figure 3:**
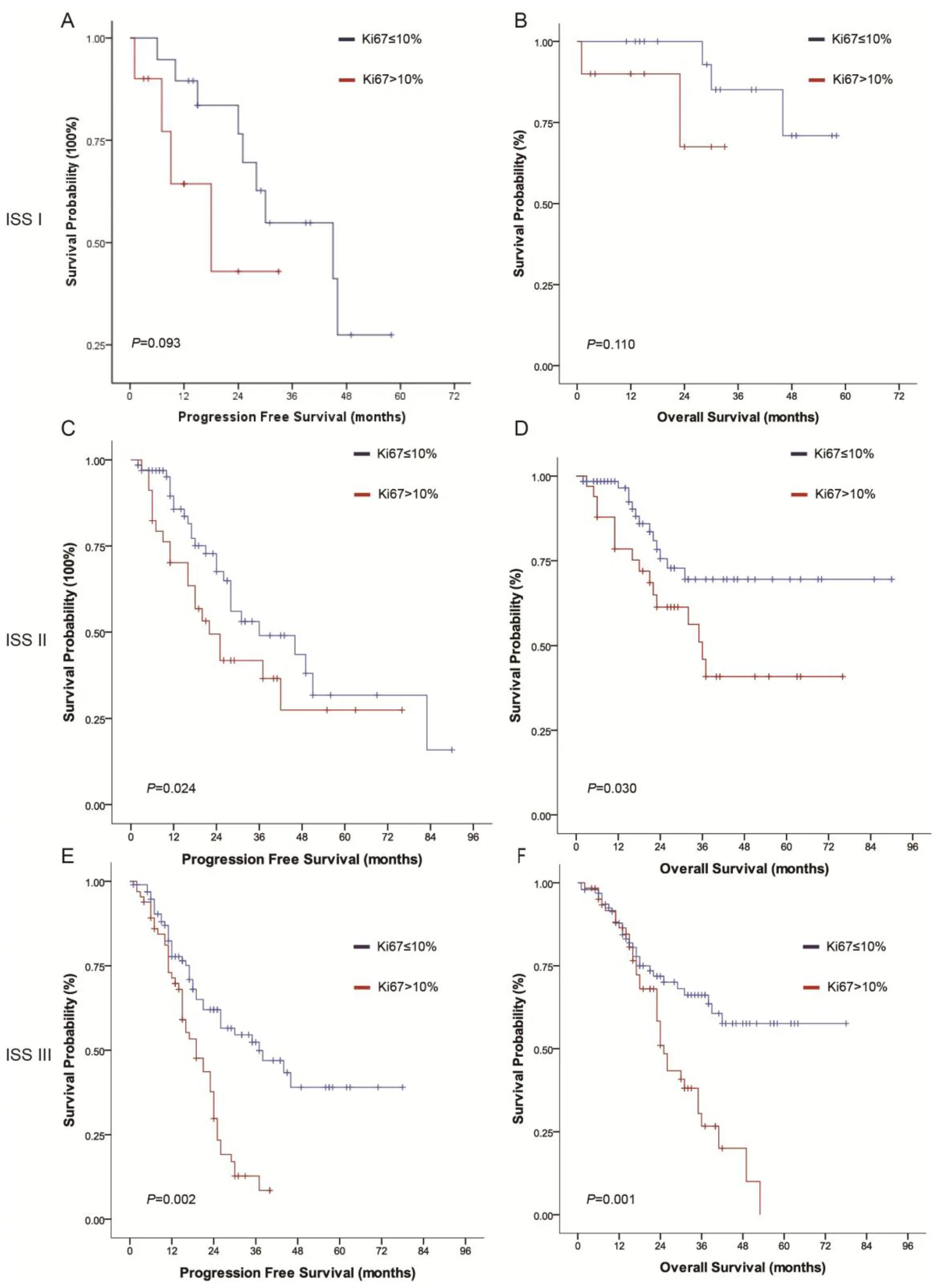
Kaplan–Meier survival curves for progression-free survival (PFS) and overall survival (OS) in ISS stage, comparing Ki 67 ≤ 10% and Ki 67 > 10% groups. (**A**) PFS and (**B**) OS in ISS I; (**C**) PFS and (**D**) OS in ISS II; (**E**) PFS and (**F**) OS in ISS III.

All 307 patients with NDMM were further subdivided within each ISS stratum according to Ki-67 proliferation index. Among patients with ISS stage I disease, the mPFS was 45.80 months (95% CI: 21.21–68.79) for those with Ki-67 ≤ 10%, versus 28.16 months (95% CI: 2.90–35.10) for patients with Ki-67 > 10%; the difference in PFS between the two subgroups did not reach statistical significance (*p* = 0.093; Figure 3A). When comparing OS in ISS stage I patients stratified by the Ki-67 cutoff, the mOS was 51.98 months (95% CI: 44.32–58.91) in the Ki-67 ≤ 10% group and 27.55 months (95% CI: 23.26–46.21) in the Ki-67 > 10% group. No statistically significant difference in OS was observed between the two subgroups (*p* = 0.110; Figure 3B).

Among patients with ISS stage II disease, we further analyzed PFS stratified by the Ki-67 index. Patients with a Ki-67 index ≤ 10% achieved a mPFS of 36.00 months (95% CI: 12.65–59.35), whereas those with a Ki-67 index > 10% had an mPFS of 21.00 months (95% CI: 13.68–30.31); the difference between those two groups was statistically significant (*p* = 0.024; Figure 3C). Consistent findings were observed for OS in the ISS stage II cohort: the mOS was 68.00 months (95% CI: 58.70–78.51) in patients with Ki-67 ≤ 10% versus 43.00 months (95% CI: 32.42–54.18) in those with Ki-67 > 10%, with a statistically significant difference (*p* = 0.030; Figure 3D).

In patients with ISS stage III disease, subgroup analysis of PFS stratified by the Ki-67 index revealed that the mPFS was 37.00 months (95% CI: 20.07–53.93) in the Ki-67 ≤ 10% subgroup and 19.00 months (95% CI: 13.40–24.60) in the Ki-67 > 10% subgroup, with a significant intergroup difference (*p* = 0.002; Figure 3E). Consistent with the PFS findings, overall survival (OS) also differed significantly between the two subgroups in ISS stage III patients (*p* = 0.001; Figure 3F). The mOS reached 53.54 months (95% CI: 46.40–60.68) in patients with Ki-67 ≤ 10%, whereas it was only 28.61 months (95% CI: 24.22–32.99) in those with Ki-67 > 10%.

### 3.6. Refinement of the ISS Staging System by Integration of the Ki-67 Proliferation Index

Patients with ISS stage I multiple myeloma were dichotomized according to Ki-67 expression levels. Although no statistically significant differences in PFS or OS were detected between the two subgroups, the cohort with Ki-67 ≤ 10% still showed favorable survival outcomes for both PFS and OS compared with the Ki-67 > 10% subgroup. In comparison, among patients stratified to ISS stages II and III, those with a Ki-67 proliferation index ≤ 10% achieved markedly longer PFS and OS than their counterparts with Ki-67 > 10%. Based on the above results, we integrated ISS staging and the Ki-67 proliferation index to establish a novel three-tiered risk stratification system for patients with NDMM. The three risk groups were defined as follows: Group I: ISS stage I with Ki-67 ≤ 10%, and ISS stage II with Ki-67 ≤ 10%; Group II: ISS stage I with Ki-67 > 10%, ISS stage II with Ki-67 > 10%, and ISS stage III with Ki-67 ≤ 10%;Group III: ISS stage III with Ki-67 > 10%.

**Figure 4:**
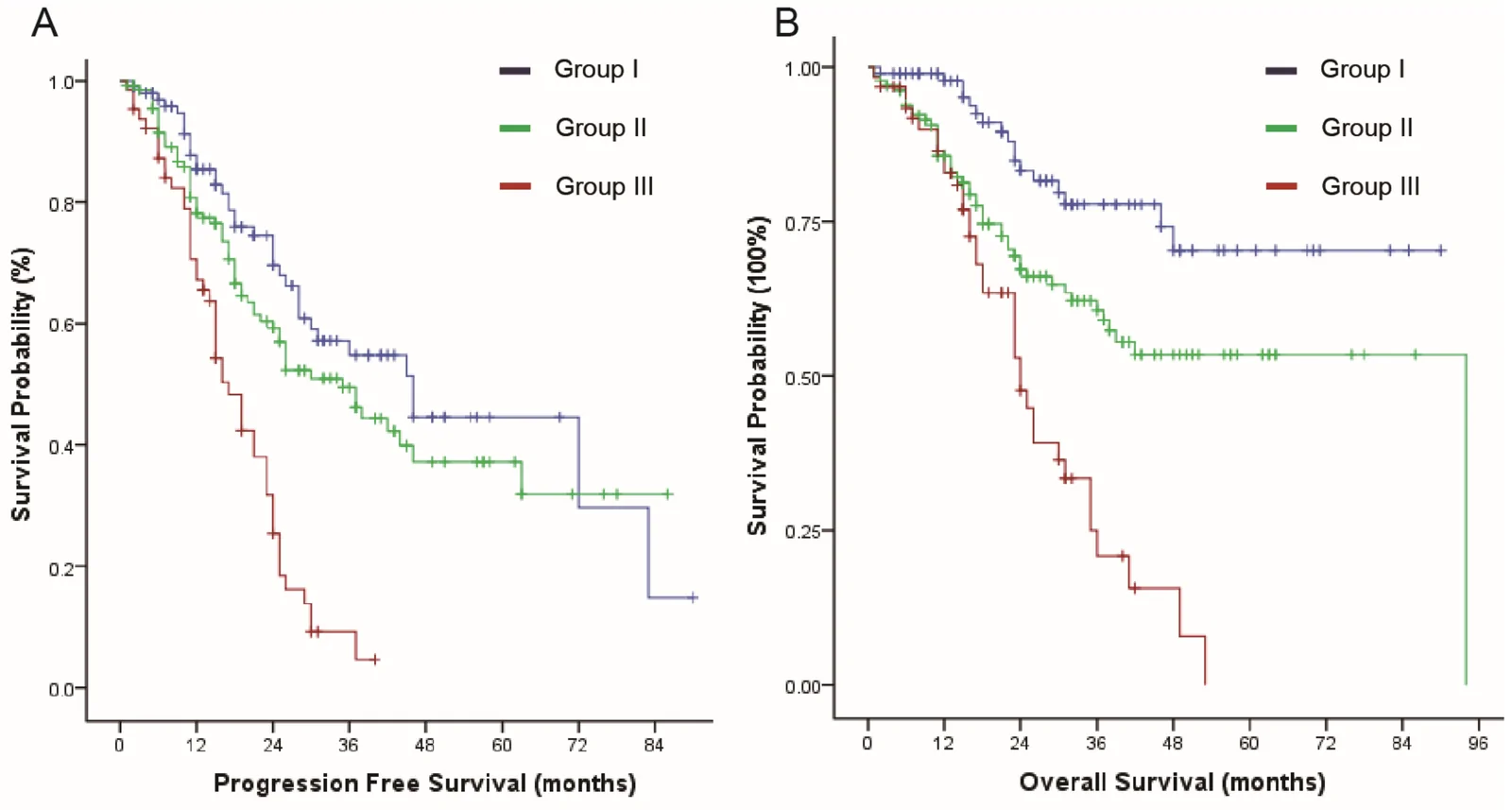
Kaplan–Meier survival curves for progression-free survival (PFS) and overall survival (OS) in the combined ISS–Ki-67 stratification model. (**A**) PFS. (**B**) OS.

A total of 307 patients with NDMM were assigned to three novel risk subgroups using the combined ISS–Ki-67 stratification model. Group I included 103 patients (33.55%), Group II included 138 patients (44.95%), and Group III included 66 patients (21.50%). Survival analyses for PFS and OS revealed robust prognostic discrimination among the three risk strata (Table 2). The mPFS was 49.74 months (95% CI: 28.81–63.19) in Group I, 35.00 months (95% CI: 24.82–45.18) in Group II, and 17.00 months (95% CI: 13.94–20.06) in Group III, with a statistically significant difference observed across the three strata (*p* = 0.011; Figure 4A). The corresponding mOS was 71.38 months (95% CI: 63.59–79.17) for Group I, 59.51 months (95% CI: 51.74–67.28) for Group II, and 26.64 months (95% CI: 22.31–30.98) for Group III. Intergroup differences in OS were also statistically significant (*p* < 0.001; Figure 4B).

## 4. Discussion

MM ranks as the second most prevalent hematological malignancy, characterized by striking biological heterogeneity, with OS ranging from mere several months to over a decade. A growing body of clinical evidence confirms that a panel of interrelated clinical and biological parameters collaboratively determine patient oncological outcomes, including age, comorbidities, cytogenetic aberrations in malignant plasma cells, the bone marrow microenvironment that supports myeloma cell proliferation, and the depth of treatment response. Identifying robust prognostic risk factors to enable precise risk stratification and facilitate the formulation of individualized therapeutic regimens remains an urgent unmet clinical challenge in myeloma research. For patients with NDMM, the ISS currently represents the most widely adopted prognostic stratification tool in global clinical practice.

This system stratifies NDMM into three prognostic subgroups based on two core serum biomarkers, namely β2-microglobulin and albumin, and delivers reliable prognostic discrimination. In the overall myeloma population, approximately 26% of patients fall into ISS stage I, 35% into stage II, and 40% into stage III. Collectively, stage II and III patients account for 75% of all cases, with stage III constituting the largest subgroup [4]. Within the MPSS staging system, patients are distributed as 19.3% ISS stage I, 36.5% stage II, and 44.2% stage III [5]. In contrast, our cohort comprised 8.79% ISS stage I, 34.20% stage II, and 57.00% stage III patients. These data indicate that the conventional ISS system assigns a substantially higher proportion of individuals in our real-world population to the high-risk stratum.

The Revised International Staging System (R-ISS) ameliorates the limitations of ISS by integrating cytogenetic aberrations and serum LDH concentrations. Nevertheless, cytogenetic testing is not universally available for all patients. The proportion of evaluable patients with complete cytogenetic profiles varies widely across published studies, ranging from 50% to 90% [6]. Even in well-designed clinical trials with comprehensive baseline data acquisition, full cytogenetic results cannot be obtained for every enrolled participant [7,8,9,10]. In real-world clinical practice, disparities in diagnostic capacity across institutions hinder routine cytogenetic testing for MM patients. In addition, CD138-positive plasma cell sorting is only accessible at a limited number of medical centers, which elevates the false-negative rate of cytogenetic assays and further reduces the proportion of patients with interpretable genetic profiles. Furthermore, a substantial subset of patients presents with concurrent myelofibrosis, accompanied by an extremely low percentage of plasma cells in bone marrow aspirates, making reliable FISH results difficult to obtain; this scenario is frequently encountered in routine clinical practice. In our retrospective cohort, complete cytogenetic data were available for only 69.38% of enrolled patients. This incomplete dataset precludes accurate risk stratification for the entire study population, as nearly one-third of patients cannot be classified using cytogenetic-integrated staging frameworks such as the R-ISS or R2-ISS.

In MM patients, the plasma cell labeling index (PCLI) quantifies the fraction of bone marrow plasma cells residing in the S phase of the cell cycle and serves as a reliable measure of the proliferative capacity of the malignant clonal plasma cells. Multiple clinical guidelines have recognized PCLI as an adverse prognostic marker in MM [11,12]. Nevertheless, widespread clinical adoption of PCLI is limited by its technically cumbersome testing workflow [13]. Ki-67 is a well-validated biomarker of cellular proliferation routinely deployed in oncopathological assessments; higher Ki-67 indices correspond to more vigorous tumor cell proliferation and a larger pool of S-phase cycling cells. Across solid malignancies, an elevated Ki-67 proliferation index consistently predicts inferior clinical outcomes [14,15]. Within hematological neoplasms, Ki-67 index also exhibits tight correlations with disease stage and overall prognosis, with higher values portending poorer survival [16,17]. Accumulating evidence has further validated the independent prognostic utility of the Ki-67 proliferation index in patients with MM.

To achieve accurate risk stratification for patients lacking cytogenetic data and improve the prognostic utility of the ISS, we combined the ISS stage with the Ki-67 proliferation index to generate refined risk subgroups in NDMM. We analyzed the association between Ki-67 index and clinical outcomes among all 307 NDMM patients and observed that patients with Ki-67 ≥ 10% exhibited significantly shorter PFS and OS compared with those with Ki-67 < 10%, indicating that an elevated Ki-67 proliferation index correlates with reduced survival duration in NDMM. These findings are consistent with a 2023 report published by Shebli Atrash, which demonstrated shorter PFS and OS in patients with high Ki-67 versus low Ki-67 expression. Their study also validated that an increased Ki-67 proliferation index constitutes an independent adverse prognostic factor for both PFS and OS in NDMM patients [18]. The underlying biological rationale lies in the fact that the Ki-67 proliferation index serves as a biomarker reflecting the proliferative activity of neoplastic plasma cells. Prior investigations have validated that Ki-67 stratifies heterogeneous clinical outcomes even among patients classified as ISS stage I [19]. However, data regarding the prognostic discriminatory value of Ki-67 specifically within ISS stage II and III cohorts remain limited.

In the established ISS–Ki-67 combined risk stratification model, Group I, Group II, and Group III accounted for 33.55%, 44.95%, and 21.50% of the overall cohort, respectively. The mPFS of the three groups was 49.74 months, 35.00 months, and 17.00 months, while the mOS was 71.38 months, 59.51 months, and 26.64 months, respectively. This integrated scoring system achieves superior prognostic discrimination by effectively identifying high-risk patients with poor outcomes within ISS stage I and distinguishing relatively low-risk cases among ISS stage III populations. Furthermore, it reduces misclassification bias, which commonly misjudges intermediate-high-risk patients as low-risk individuals or misclassifies low-intermediate-risk patients as high-risk individuals. Such refined risk stratification enables precise prognostic evaluation at initial diagnosis, thereby assisting clinicians in delivering risk-adapted and individualized therapeutic strategies for NDMM patients.

Admittedly, the combined Ki-67-ISS stratification model has some limitations. Regarding Ki-67 measurement and calculation, our assay cannot reliably distinguish plasma cells from non-plasma-cell populations, and interference from other cell components cannot be fully eliminated. In recent years, several research centers have applied CD138/Ki-67 double-staining immunohistochemistry to specifically assess the Ki-67 proliferation index in plasma cells, which may further improve measurement accuracy for patients with multiple myeloma (MM). At the same time, this is a single-center retrospective analysis, which is inherently subject to selection bias. All enrolled patients were recruited from one medical institution, and external multi-center validation cohorts are absent; hence, our combined Ki-67-ISS stratification model requires further verification in independent real-world cohorts.

## 5. Conclusions

NDMM patients with Ki-67 proliferation index > 10% have significantly worse prognosis than those with Ki-67 ≤ 10%. A high Ki-67 index (>10%) is identified as an independent adverse prognostic parameter for NDMM. Furthermore, the integration of the Ki-67 proliferation index with ISS staging can substantially enhance the accuracy of prognostic risk stratification in patients with NDMM.

## Figures and Tables

**Table 1:** Comparison of baseline characteristics among patients with newly diagnosed multiple myeloma in different Ki67 groups.

Clinical Features	Ki67 > 10% (n = 108)	Ki67 ≤ 10% (n = 199)	*p* Value
Sex, [n (%)]			0.643
Male	63 (58.33)	118 (62.04)	
Female	45 (41.67)	81 (37.96)	
Age [years, M (range)]	60 (39–80)	61 (37–86)	0.899
M Protein Type [n (%)]			0.655
IgG	53 (49.07)	81 (40.70)	
IgA	20 (18.52)	46 (23.12)	
IgD	5 (4.63)	10 (5.03)	
Light chain type	26 (24.07)	56 (28.14)	
Non-secretory type	4 (3.70)	5 (2.51)	
Biclonal	0 (0.00)	1 (0.50)	
Durie–Salmon stage, n (%)			0.923
Stage I	5 (4.63)	8 (4.02)	
Stage II	9 (8.33)	17 (8.54)	
Stage III	94 (87.04)	174 (87.44)	
ISS stage, n (%)			
Stage I	8 (7.41)	19 (9.55)	0.579
Stage II	34 (31.48)	71 (35.68)	
Stage III	66 (61.11)	109 (54.77)	
Alb [g/L, M (range)]	32.98 (21.80–45.17)	31.61 (20.16–41.27)	0.139
Serum creatinine, n (%)			0.780
≥177 μmol/L	35 (32.41)	61 (30.65)	
<177 μmol/L	73 (67.59)	138 (69.35)	
HGB [g/L, M (range)]	85 (51–148)	91 (47–152)	0.041
PLT [×10^9^/L, M (range)]	129 (15–328)	183 (17–332)	0.015
LDH [U/L, M (range)]	212 (55–571)	287 (71–748)	0.265
Serum calcium [mmol/L, M (range)]	2.38 (1.71–3.12)	2.34 (1.69–3.81)	0.547
Plasma cell percentage in bone marrow smear [M (range)]	29.8 (10.5–91.5)	34.0 (12.5–95.0)	0.107
1q21 amplification, n/N (%)	43/82 (52.44)	52/131 (39.69)	0.069
del(17p), n/N (%)	13/80 (16.25)	5/128 (3.91)	0.002
del(13q), n/N (%)	28/80 (35.00)	36/129 (27.91)	0.280

**Table 2:** Comparison of ISS staging and ISS combined with Ki67 staging.

Stage	ISS (n/N%)	ISS + Ki-67 (n/N%)
I	27/307 (8.79)	103/307 (33.55)
II	105/307 (34.20)	138/307 (44.95)
III	175/307 (57.00)	66/307 (21.50)

## Data Availability

Due to institutional privacy policy requirements, deidentified individual participant data that underlie the results reported in this article, including text, tables, figures, and appendices, will be made available upon reasonable request to the corresponding author (qinxiaoqi6@163.com), beginning 3 months and ending 5 years after publication.

## Abbreviations

The following abbreviations are used in this manuscript:

NDMM	newly diagnosed multiple myeloma
ISS	International Staging System
mPFS	median progression-free survival
mOS	median Overall Survival
LDH	lactate dehydrogenase
R-ISS	Revised International Staging System
R2-ISS	Second Revision of the International Staging System
MASS	Mayo Additive Staging System
MPSS	Myeloma Prognostic Score System
PCLI	plasma cell labeling index
